# Case Report: A case of synchronous right upper lobe adenocarcinoma and left lower lobe squamous cell carcinoma treated with immune checkpoint inhibitor plus chemotherapy

**DOI:** 10.3389/fonc.2023.1062138

**Published:** 2023-01-25

**Authors:** Yujiao Liu, Han Yu, Youhong Dong, Dongdong Zhang

**Affiliations:** ^1^ State Key Laboratory of Separation Membranes and Membrane Processes, School of Chemistry, Tiangong University, Tianjin, China; ^2^ Department of Pathology, Xiangyang No.1 People’s Hospital, Hubei University of Medicine, Xiangyang, China; ^3^ Department of Oncology, Xiangyang No. 1 People’s Hospital, Hubei University of Medicine, Xiangyang, China

**Keywords:** multiple primary lung cancers, intrapulmonary metastases, squamous cell carcinoma, adenocarcinoma, synchronous MPLC

## Abstract

Globally, lung cancer is the leading cause of cancer-related mortality. Multiple primary lung cancers (MPLC) account for a very small portion of all primary lung cancer cases. Importantly, a quick and precise differentiation between MPLC and intrapulmonary metastases is directly related to patient prognoses as treatment strategies vary according to pathological type. Synchronous MPLC are most commonly seen in the same lung. Here, we report a rare case of a patient with synchronous MPLC of both lungs. A 67-year-old man, with a 1-month cough and expectoration history, was admitted in our hospital. Computed tomography (CT) chest scan revealed a lower lobe nodule in the left lung and an upper lobe nodule in the right lung. He underwent successive fiberoptic bronchoscopy and CT-guided percutaneous pulmonary aspiration biopsy of both lungs. The pathological diagnosis was squamous cell carcinoma of the left lung and adenocarcinoma of the right lung.

## Introduction

An early diagnosis and the increasing effectiveness of cancer therapies have significantly prolonged overall survival times in cancer patients. However, cancer survivors had a higher risk of developing new malignancies when compared with the general population. This situation poses new problems which are manifested as soaring incidences of multiple primary tumors (1).

Globally, lung cancer is the most common cause of cancer death, with the lungs one of the most common sites in terms of multiple primary malignancy (2). Initial diagnostic criteria for “multiple primary lung cancers” (MPLC) were published in 1975 based on histology and tumor locations (3). However, these criteria could not differentiate MPLC from intrapulmonary metastases (IPM). Furthermore, special cases may be misdiagnosed as MPLC without the pathological confirmation of every lesion, such as lepidic adenocarcinoma, it displays multiple pure ground-glass opacity lesions by computed tomography (CT) (4). In 2015, the World Health Organization Classification of Tumors of the Lung redefined MPLC diagnostic criteria and recommended a multidisciplinary tumor board approach to confirm the diagnosis (5). In addition to histological subtyping, algorithms based on comprehensive clinical and imaging variables and comparative genomic hybridization array information, were also applied to differentiate MPLC from IPM (6, 7).

MPLC is subdivided into two categories depending on the time of diagnosis of each primary site; metachronous MPLC (MMPLC) and synchronous MPLC (SMPLC). MMPLC is common and generally occurs in sequence after more than a 2-year cancer-free interval, while SMPLC is relatively rare and presents simultaneously or within a six months interval (8). In this study, we present a SMPLC case with right upper lobe adenocarcinoma and left lower lobe squamous cell carcinoma.

## Case presentation

**Figure 1:**
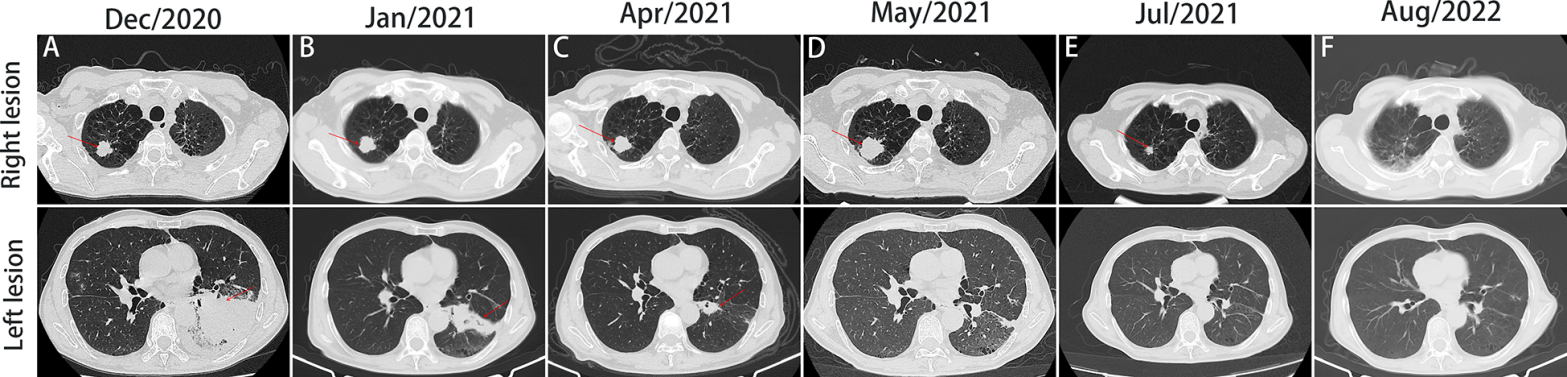
Computed tomography (CT) scan of the patient throughout the whole course of diagnosis and treatment. Figures A-E, imaging changes of chest CT (mediastinal window and pulmonary window) at different times.

**Figure 2:**
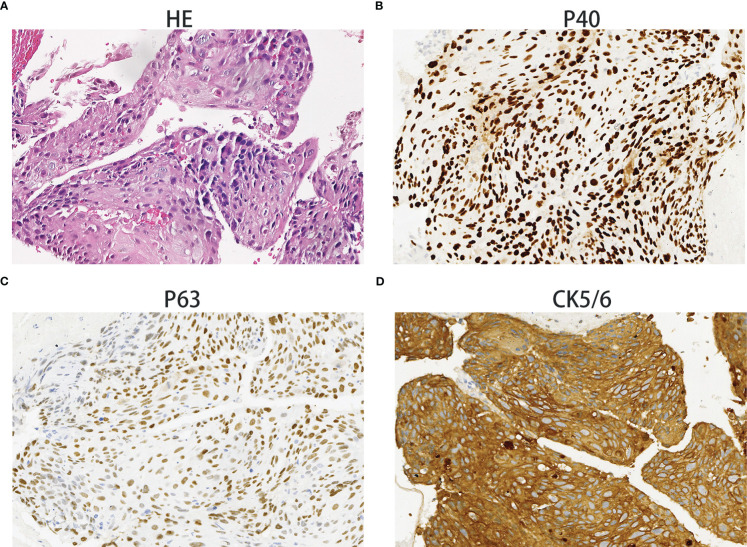
Squamous cell carcinoma. Hematoxylin and eosin staining showing squamous cell carcinoma histology **(A)**. The immunohistochemical examination indicated malignant cells immunoreactive for P40 **(B)**; positive for P63 **(C)**; strongly positive for CK5/6 **(D)**. Magnification 100×.

**Figure 3:**
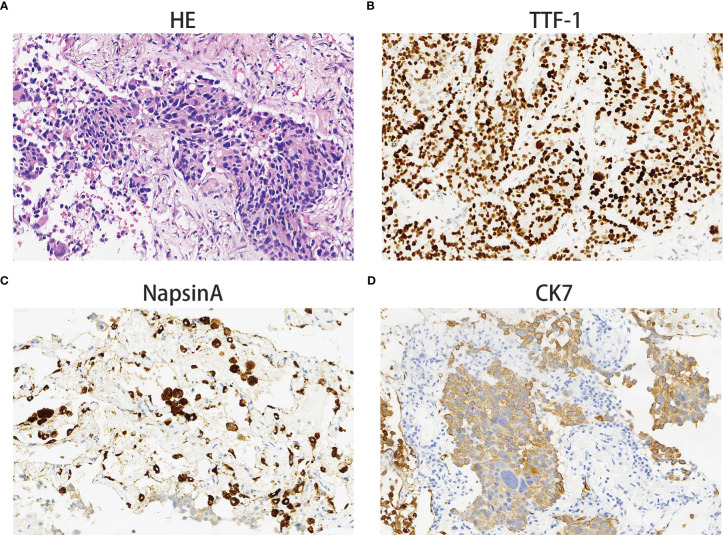
Adenocarcinoma. Hematoxylin and eosin staining showing adenocarcinoma histology **(A)**. The immunohistochemical examination indicated malignant cells immunoreactive for TTF-1 **(B)**; weakly positive for NapsinA **(C)**; strongly positive for CK7 **(D)**. Magnification 100×.

**Figure 4:**
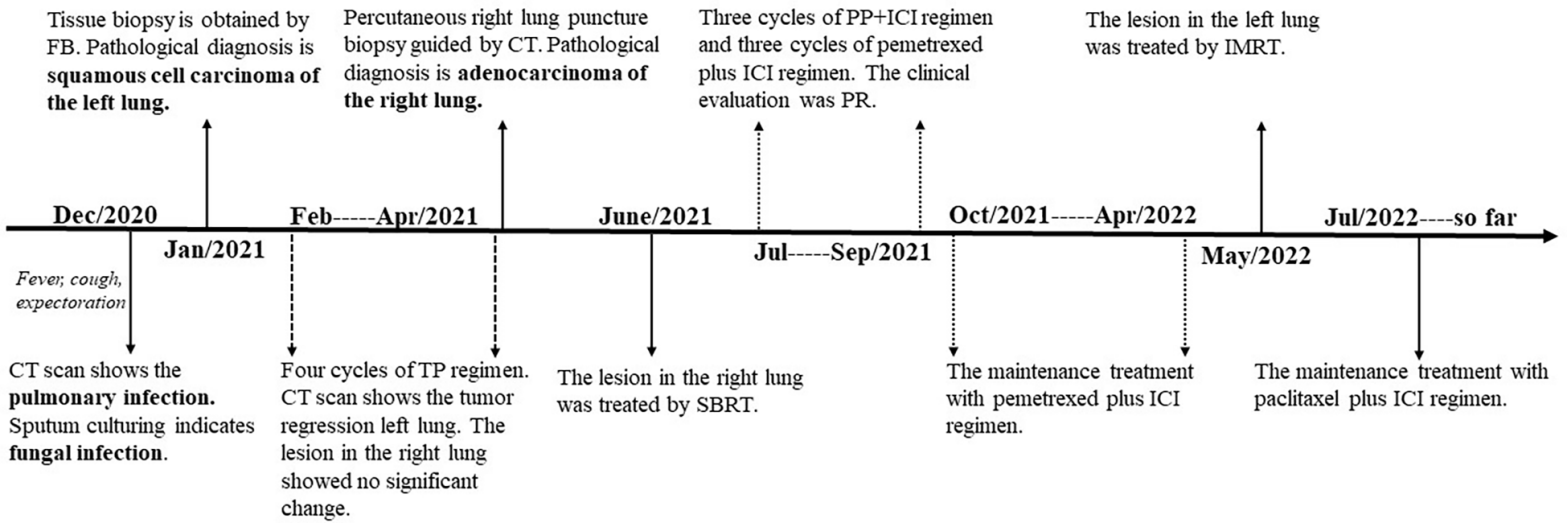
The timeline of the patient’s events from diagnosis to treatment. CT, computed tomography; FB, fiberoptic bronchoscopy; TP, docetaxel plus nedaplatin; SBRT, stereotactic body radiation therapy; ICI, immune checkpoint inhibitor; PP, pemetrexed plus carboplatin; PR, partial remission; IMRT, intensity-modulated radiation therapy.

On December 28^th^, 2020, a 67-year-old male came to our institute with a 1-month history of fever, cough and expectoration. He had smoked for 20 years. A physical examination revealed moist rales in the left lung base. From routine blood tests, the white blood cell count was 18.68×10^9^/L. Inflammatory indicators, such as C-reactive protein and procalcitonin, were significantly elevated. Tumor marker detection indicated carcinoembryonic antigen serum levels were slightly elevated. Sputum cultures suggested a fungal infection. CT scan identified an infectious lesion in the left lower lobe, accompanied by pulmonary atelectasis and an upper lobe nodule in the right lung (**Figure 1A**). He then underwent fiberoptic bronchoscopy (FB). The pathological diagnosis from this was squamous cell carcinoma of the left lung. Hematoxylin and eosin (HE) staining showed squamous cell carcinoma (**Figure 2A**). Immumohistochemical staining (IHC) showed a P40, P63, and CK5/6 positive status (**Figures 2B–D**). Considering a fungal infection in the left lower lobe and atelectasis, he first received anti-infection therapy for approximately 1 month. The patient was then admitted and reviewed on January 23^rd^, 2021, a chest CT indicated that the soft tissue mass and obstructive atelectasis in the left lower lobe were absorbed (**Figure 1B**). Considering bilateral lung metastases before treatment, he received adjuvant chemotherapy as an initial treatment. After four cycles of docetaxel plus nedaplatin (TP) regimen, CT showed a partial response in the left lower lobe; however, the right lung nodule showed no significant change (**Figure 1C**). To make a definite diagnosis of the right lung lesion and inaugurate timely and responsive clinical treatment, he underwent a percutaneous right lung puncture biopsy guided by CT. Pathological examination showed changes which were completely different from the left lung (**Figure 3A**). IHC staining showed that CK7 and TTF-1 were strongly positive, while NapsinA was weakly positive (**Figures 3B–D**), therefore, the pathological diagnosis was adenocarcinoma. Genetic testing indicated a *TP53* mutation, *EGFR* was wild type, and the tumor cell proportion score was 5%. After completion of the 5^th^ TP regimen cycle on May 14^th^, 2021, CT showed a very good partial response in the left lower lobe, while the right lung nodular was larger than before (**Figure 1D**). Considering the significant regression of the left lung lesion, the patient commenced adenocarcinoma treatment. He received stereotactic radiotherapy on June 14^th^, 2021, the prescribed dose was 60.0 Gy in eight fractions. He subsequently received immune checkpoint inhibitor (ICI) therapy in combination with pemetrexed and carboplatin (PP). The right lung lesion had obviously diminished after completion of the first immunochemotherapy cycle (**Figure 1E**) and he achieved very good remission in both lungs after three cycles of this regimen (**Figure 1F**). Due to the frequency of grade III-IV myelosuppression and pulmonary infection, he was given another three cycles of pemetrexed plus ICI, followed by intensity modulated radiation therapy for the left lung lesion. The prescribed pulmonary dose was 60.0 Gy, with daily fractions of 2.0 Gy. Finally, paclitaxel plus ICI was administrated every 3 weeks for maintenance treatment. Currently, the patient exhibits no evidence of disease recurrence and progression. Detailed diagnosis information and a treatment flow chart are shown (**Figure 4**).

## Discussion

We report a patient with SMPLC who was successively diagnosed with left lung squamous cell carcinoma and right lung adenocarcinoma. SMPLC were identified in 1924 and then were widely recognized. The estimated SMPLC incidence accounts for 0.2%–8% of all lung cancers and has steadily increased over the past three decades (9).

MPLC may be caused by intrinsic and non-intrinsic cancer risk factor; intrinsic risk factors are defined as the genetic mutations caused by DNA replication errors, including *EGFR, KRAS, TP53*, or *PARP1* mutations (10). Non-intrinsic risk factors refer to modified endogenous factors, including lifestyle, radiation, and any other endogenous factors. It is accepted that smoking and the widespread use of high-resolution CT and positron emission tomography-CT contribute to MPLC occurrence (9).

Currently, no golden diagnostic criteria exist for MPLC due to the tumor heterogeneity and a poor understanding of associated clinicopathological characteristics. Therefore, a diagnosis should be considered by a multidisciplinary tumor board based on clinical manifestations, imaging features, pathological characteristics, and molecular genetic characteristics (11). MPLC stage classification is critical for patients, because staging affects initial treatment choices. For MMPLC, the second tumor should be staged as the primary according to the 7th Tumor-Node-Metastasis (TNM) classification guidelines. However, SMPLC staging is ambiguous, each tumor should be staged separately and only one TNM stage should be provided based on all combined tumors (8). In some situations, SMPLC is defined as the highest pathological stage (12).

Surgery is the cornerstone treatment for early MPLC without lymph node involvement. For surgery, the indications and contraindications must be strictly understood. Tumor’s size and location, Eastern Cooperative Oncology Group performance status, and cardiopulmonary function must be considered before surgery. Operational styles are also closely related to patient quality of life and prognosis. Pneumonectomy has a high risk of postoperative respiratory failure and may herald a poor prognosis. Though segmentectomy or wedge resection has a risk of local recurrence (up to 15%), it is tolerable for patients with compromised pulmonary function who are unfit for more extensive resection (13). The Lung Study Group recommends that pneumonectomy should be avoided whenever possible, thus limited resection remains the mainstay treatment for MPLC.

Systemic chemotherapy is crucial for patients with mediastinal lymph node and distant metastasis. For patients with MMPLC, the second tumor should be treated as a primary tumor. However, the SMPLC treatment strategy is that each tumor should be staged and treated separately (14). Due to differences in chemotherapy regimens between adenocarcinoma and squamous cell carcinoma, it is often difficult for clinicians to select which regimen should be first perform for SMPLC patients with distinct tumor histology. It remains to be determined whether both effective regimen or sequential treatment for each tumor should be the first choice. In our study, the patient benefited from sequential treatments. After five TP regimen cycles, the lesion shrank and obstructive pneumonia improved significantly in this lung. Then, the right lung nodule significantly was reduced after the replacement of the PP regimen. Our strategy indicated that sequential treatment was effective for patients with SMPLC in both lungs with different pathological types, especially when the problem was more severe in one lung, such as severe pulmonary infection, obstructive pneumonia or pulmonary atelectasis.

Previous studies reported that ICI plus chemotherapy generated higher objective remission rates and superior overall survival and progression-free survival rates in patients with previously untreated, metastatic, non-small cell lung cancer (NSCLC) when compared with single chemotherapy (15, 16). Our patient benefited from combined ICI and chemotherapy in adjuvant and maintenance treatment phases. Thus, ICI may be recommended for patients with advanced MPLC, but this hypothesis requires further research.

Radiotherapy improves local control rates in cancer. Intensity- modulated radiation therapy or three-dimensional conformal radiation is still an important treatment strategy for stage I–IIIB inoperable patients with lung cancers (17). Several studies reported that stereotactic body radiation therapy (SBRT) generated similar clinical outcomes in early NSCLC when compared with surgical treatment (18). Furthermore, SBRT may be a potential cure approach for early stage MPLC as it achieves promising long-term tumor control and survival (19). Given these factors, radiation is indispensable for MPLC treatment.

The 5-year overall survival for MPLC ranges from 20% to 70% (12). The prognosis is related to several clinical factors. Patients with same tumor histology are relatively favorable when compared with those with different histology (20). Patients with SMPLC or early MMPLC have lower survival rates when compared with patients with late MMPLC (21). Pneumonectomy has a higher early postoperative mortality rate and a shorter overall survival rate when compared with limited resection (21). Multivariable analyses have indicated that pathological stage and lymph node metastases are associated with prognosis (21). Therefore, the MPLC diagnosis and treatment should be determined by a multi-disciplinary team.

In conclusion, we reported the rare but interesting case of a patient with SMPLC in both lungs. The patient was first misdiagnosed with squamous cell carcinoma of the left lung, accompanied by right IPM and mediastinal lymph node metastasis. He received sequential chemotherapy plus ICI and radiotherapy without surgical resection, and is doing well under ICI maintenance. We believe this interesting case may provoke debate in the literature about the precise diagnosis and effective treatment for MPLC cases. Our result also indicated that the proper tumor subclassification (with IHC and molecular methods) was important for patients with SMPLC to receive individualize treatment and achieve better outcome.

## Data availability statement

The original contributions presented in the study are included in the article/Supplementary Material. Further inquiries can be directed to the corresponding author.

## Ethics statement

This study was approved by the Ethics and Scientific Committee of Hubei University of Medicine with approval number XYY2021002. The patients/participants provided their written informed consent to participate in this study. Written informed consent was obtained from the individual for the publication of any potentially identifiable images or data included in this article.​

## Author contributions

Conceptualization, DZ; data curation and writing, writing—review and editing, YL, YD, and HY; funding acquisition, DZ. All authors contributed to the article and approved the submitted version.
